# Content, Structure and Delivery Characteristics of Yoga Interventions for the Management of Osteoarthritis: A Systematic Review Protocol

**DOI:** 10.3390/ijerph19105806

**Published:** 2022-05-10

**Authors:** Isha Biswas, Sarah Lewis, Kaushik Chattopadhyay

**Affiliations:** 1 Lifespan and Population Health Academic Unit, School of Medicine, University of Nottingham, Nottingham NG5 1PB, UK; sarah.lewis@nottingham.ac.uk (S.L.); kaushik.chattopadhyay@nottingham.ac.uk (K.C.); 2 The Nottingham Centre for Evidence-Based Healthcare: A JBI Centre of Excellence, Nottingham NG5 1PB, UK

**Keywords:** osteoarthritis, management, yoga, systematic review

## Abstract

The global burden of osteoarthritis among adults is rising. Yoga might be a potential solution for the management of osteoarthritis. This systematic review aims to synthesise the content, structure and delivery characteristics of effective yoga interventions for the management of osteoarthritis. The JBI methodology for systematic reviews of effectiveness and the Preferred Reporting Items for Systematic Reviews and Meta-analyses (PRISMA) guidelines will be followed. Randomised controlled trials (RCTs) assessing the effectiveness of yoga interventions for the management of osteoarthritis in adults will be included in this review. We aim to search the following databases to find published and unpublished studies: MEDLINE, EMBASE, CINAHL, PsycInfo, SPORTDiscus, AMED, Web of Science, CENTRAL, TRIP, AYUSH Research Portal, ABIM, CAM-QUEST, PeDro, OpenGrey, EthOS, ProQuest Dissertations and Theses and DART-Europe-e-theses portal. No date or language restrictions will be applied. A narrative synthesis will be conducted with the help of tables. A meta-regression will be conducted to explore the statistical evidence for which the components (content, structure and delivery characteristics) of yoga interventions are effective.

## 1. Introduction

### 1.1. Osteoarthritis

Osteoarthritis is a long-term condition of the joints with symptoms such as pain, stiffness and difficulty in movement [1]. It is the most common type of arthritis in the world [1,2]. It erodes the cartilage lining of the joints, causing joint deformities [1]. Traditionally, it is considered to be “non-inflammatory” [2]. However, evolving research has found inflammatory mediators to be involved in the onset and progress of the condition, making it difficult to classify osteoarthritis as inflammatory or non-inflammatory [3]. It can affect any joint, but the most common ones affected are the knees, hips and small joints of the hands [1]. The diagnosis of osteoarthritis is based on physical examination, radiographic and magnetic resonance imaging (MRI) findings and/or arthroscopy [1,4]. Radiographic findings do not always correlate with the clinical severity of osteoarthritis [5]. Some of the comorbidities associated with osteoarthritis are diabetes, cardiovascular diseases, hypertension, metabolic syndrome, stroke, back pain and depression [6,7,8].

### 1.2. Types and Risk Factors of Osteoarthritis

Osteoarthritis can be classified into two types—primary and secondary [2]. Primary osteoarthritis is the most common type and is not caused by a pre-existing health condition but is associated with some risk factors [2]. The risk factors for osteoarthritis include sociodemographic factors, such as increasing age, female sex and ethnicity (globally, Black/African-American people are more prone to knee osteoarthritis compared to Non-Hispanic White/Caucasian people); lifestyle factors such as a lack of physical activity; genetic factors; health conditions, such as obesity, muscle weakness and joint injury (from physically strenuous occupation and sports activities); and nutritional deficiencies, such as a deficiency of vitamin D, vitamin C and vitamin K [1,9,10,11]. Secondary osteoarthritis occurs due to a pre-existing health condition, including but not limited to, joint abnormality, any other type of arthritis (such as rheumatoid arthritis and gout) and osteoporosis [1,2].

### 1.3. Global Burden of Osteoarthritis

Osteoarthritis poses tremendous health (physical and psychological), social and economic burden [12,13,14,15,16,17,18]. Some of the physical health consequences of osteoarthritis include its symptoms, such as joint pain and stiffness, difficulty in movement and occurrences of falls [13,14]. Some common psychological health consequences are anxiety and depression [12]. Social consequences include withdrawal from social participation and occupational activities [13]. The economic impact of osteoarthritis includes huge direct and indirect costs [14,16]. Direct costs arise due to medical and surgical treatments such as knee replacement surgery [17,18]. Indirect costs arise due to absenteeism from work and the loss of work productivity [16]. Reduced self-efficacy and poor quality of life are some other issues [15].

In terms of numbers and percentages, about 3.3–3.6% of the global population is affected by osteoarthritis, and it causes moderate-to-severe disability, making it the 11th most debilitating disease in the world [2]. Osteoarthritis contributes to 18.9 million years lived with disability (YLDs), which is 2.2% of the total global YLDs [2]. Osteoarthritis of the knee, hip, hand and other joints (e.g., foot, shoulder and wrist) contribute to 60.9%, 5.5%, 23.5% and 10.2% of global YLDs, respectively [2]. The global disability-adjusted life years (DALYs) related to osteoarthritis are high and rising [19].

### 1.4. Current Management Strategies of Osteoarthritis and Their Limitations

The main aim of osteoarthritis management is to minimise joint pain and the loss of function [20]. It is managed using pharmacological and non-pharmacological interventions, as well as surgical interventions in severe cases [20]. Pharmacological interventions include oral, topical and/or intraarticular options [2]. Non-steroidal anti-inflammatory drugs (NSAIDs), prescribed orally (e.g., ibuprofen), are the first line of treatment but have side effects, such as gastrointestinal toxicity and cardiovascular effects [2]. Topical NSAIDs (e.g., diclofenac gel) are less effective than their oral counterparts and have fewer gastrointestinal and other systemic side effects; however, they often cause local skin irritation [21]. Intra-articular corticosteroid injections provide short-term relief from pain and improve function, but using them more than once every four months can result in cartilage and joint damage [22]. The side effects and costs associated with the use of pharmacological interventions are some of the reasons why people with osteoarthritis might be reluctant to use them [20,22]. Along with pharmacological interventions, the use of non-pharmacological interventions, such as exercise to improve muscle strength (e.g., the hamstring muscle in the case of knee osteoarthritis) and weight loss for overweight and obese individuals, are recommended [23]. Surgical treatments such as joint replacements are needed when other treatments have not been effective or in cases of severe joint damage [2].

### 1.5. Yoga: A Potential Solution for the Management of Osteoarthritis

Yoga is an ancient practice, with origins in the Indian subcontinent, that aims to offer a holistic sense of well-being of the body and mind [24]. Yoga philosophy and practice were first described by Patanjali in the classic text *Yoga Sutras* [25]. The multi-factorial approach of yoga includes components such as yogic poses (asana), breathing practices (pranayama) and meditation (dhyana) and relaxation practices, along with moderation in lifestyle [25]. Among the seven major branches of yoga, Hatha yoga is the most popular [26]. There are various styles of Hatha yoga, and each has its distinct emphasis on the individual components [26]. Yoga is becoming popular across the world, and there are about 300 million people who practice yoga globally [27]. Generally, yoga is easy to learn and safe to practice, demands a low-to-moderate level of supervision, is inexpensive to maintain because of the minimal equipment requirement and can be practised indoors and outdoors [28,29]. 

Several systematic reviews and meta-analyses have reported the beneficial effects of yoga interventions on osteoarthritis outcomes, such as pain relief and functional improvement [30,31,32,33,34,35]. These reviews have included randomised controlled trials (RCTs) [30,31,32,33,34], except one, which also included other study designs [35]. This recent systematic review and meta-analysis of 20 RCTs and 2 case series on knee and hip osteoarthritis showed that yoga significantly improved pain (mean difference (MD) −1.82, 95% confidence interval (CI) −2.96 to −0.67) and physical function (−6.07, −9.75 to −2.39) compared to no intervention or usual care [35]. No adverse events related to yoga were reported [32]. 

The beneficial effects of yoga on osteoarthritis outcomes can be explained by some potential mechanisms. Yoga practice generally begins with slow movement sequences to increase blood flow and warm up the muscles [36]. This is followed by holding certain yogic poses, including flexion, extension, adduction, abduction and rotation, which engage the muscles in isometric contraction [36,37,38]. The movement of joints increases flexibility, whereas standing yogic poses improve balance and coordination by strengthening major muscle groups (e.g., hamstring muscles and quads) [39,40,41]. This might lead to a reduction in pain and stiffness and improved function [41,42,43]. There is some evidence that shows that the practice of specific yogic poses and breath control practices improve the process of respiration and calm the mind by reducing stress, anxiety and depression [43,44,45]. That is, yoga potentially provides physical health benefits; reduces stress, anxiety and depression; and enhances self-esteem and quality of life [30]. 

### 1.6. The Rationale for the Systematic Review

All the above-mentioned systematic reviews have only described but not synthesised the content, structure and delivery characteristics of yoga interventions for the management of osteoarthritis [30,31,32,33,34,35]. Moreover, a meta-regression has never been conducted to explore the statistical evidence for which the components of yoga interventions (e.g., content, structure and delivery characteristics) are effective. Thus, there is a need to conduct such a systematic review so that the content, structure and delivery characteristics of effective yoga interventions for the management of osteoarthritis can be synthesised and used in future research and practice.

### 1.7. Aim

The aim of this systematic review is to synthesise the content, structure and delivery characteristics of effective yoga interventions for the management of osteoarthritis.

## 2. Methods

This systematic review will be conducted in accordance with the JBI methodology for systematic reviews of effectiveness and the Preferred Reporting Items for Systematic Reviews and Meta-analyses (PRISMA) guidelines [46,47]. The systematic review protocol is registered with PROSPERO (CRD42022298155).

### 2.1. Inclusion Criteria

#### 2.1.1. Population

This systematic review will include studies conducted among adults (aged ≥18 years) diagnosed with osteoarthritis of one or more joints. No restrictions will be applied regarding the diagnostic criteria of osteoarthritis, and to name a few, diagnoses based on physical examination, radiographic and MRI findings and/or arthroscopy will be included. If a study includes both children and adults, only the relevant information about adults will be extracted. If it is not possible to extract the relevant information about adults, the study will be excluded.

#### 2.1.2. Intervention

Studies reporting at least one of the major components of yoga, namely, asana (yogic poses), pranayama (breathing practices) and dhyana (meditation) and relaxation practices, will be included. There will be no restrictions on the type, frequency, duration and delivery mode of the yoga intervention. Studies that include multimodal interventions (which include yoga among other interventions) will be excluded if relevant data cannot be extracted. Studies will also be excluded if they did not explicitly label the intervention as yoga.

#### 2.1.3. Comparator

Studies comparing yoga interventions with no intervention, sham intervention, non-pharmaceutical intervention (e.g., diet, physical activity and educational intervention) or pharmaceutical intervention (e.g., NSAIDs) will be included. Studies with co-interventions will be included as long as all the eligible study groups were allowed to do so. Studies with a head-to-head comparison of two or more yoga interventions (i.e., different in terms of content, structure or delivery characteristics) will be excluded.

#### 2.1.4. Outcome

This systematic review will include studies that assessed the core outcomes of osteoarthritis, i.e., pain and function, as recommended by several guidelines [48,49,50,51,52]. Pain assessed using any scale will be eligible (e.g., visual analogue scale (VAS) and numeric rating scale (NRS)), and function assessed using any scale will be eligible (e.g., arthritis impact measurement Scale (AIMS), any joint-specific scale such as Foot and Ankle Ability Measure (FAAM), Knee Injury and Osteoarthritis Outcome Score (KOOS) and Hip Disability and Osteoarthritis Outcomes Survey (HOOS)) [51,52]. Radiographic outcomes, such as joint space narrowing and osteocyte formation, will not be included in this review, as the inter- and intra-observer variabilities in interpreting radiographs may affect the specificity of the classification criteria and are not standardised to be considered as core outcomes [48,51].

#### 2.1.5. Study Design

Considering the feasibility and practicality of the proposed work and the hierarchy of study designs, only RCTs will be included in this systematic review.

### 2.2. Data Sources and Search Strategies

The following 13 databases will be searched from their inception dates to find published studies: (i) MEDLINE (from 1946; Ovid), (ii) EMBASE (from 1974; Ovid), (iii) CINAHL (from 1994; EBSCOHost), (iv) PsycInfo (from 1806; Ovid), (v) SPORTDiscus (from 2004; EBSCOhost), (vi) Allied and Complementary Medicine (AMED) (from 1985; Ovid), (vii) Web of Science (from 1900; Clarivate analytics), (viii) Cochrane Central Register of Controlled Trials (CENTRAL) (from 1996), (ix) Turning Research Into Practice (TRIP) (from 2014), (x) AYUSH Research Portal (http://ayushportal.nic.in/, accessed on 21 January 2022), (xi) A Bibliography of Indian Medicine (ABIM) (http://indianmedicine.eldoc.ub.rug.nl/, accessed on 21 January 2022), (xii) CAM-QUEST (https://www.cam-quest.org/en, accessed on 21 January 2022) and (xiii) Physiotherapy Evidence Database (PeDro) (from 1999). Unpublished studies will be searched using (i) OpenGrey (from 1997), (ii) EthOS (from 1925), (iii) ProQuest Dissertations and Theses (from 1980) and (iv) DART-Europe-e-theses portal (from 1999). The reference list of all the included studies and relevant previous systematic reviews will be screened for additional studies.

The search strategies are developed based on the following and in consultation with a Research Librarian at the University of Nottingham: (i) the yoga component is based on a previous relevant systematic review protocol [53], (ii) the osteoarthritis component is based on the search strategies reported in the UK’s National Institute for Health and Care Excellence (NICE) guidelines for the management of osteoarthritis [54] and existing Cochrane systematic reviews on osteoarthritis [55,56], and (iii) the pre-designed search filters for RCTs are used [57,58,59]. All the search strategies are detailed in Appendix A. No date or language restrictions will be applied.

### 2.3. Study Screening and Selection

All the identified citations will be collated and uploaded onto Endnote X9 (Clarivate Analytics, PA, USA) [60], and duplicates will be removed. The remaining records will then be imported into Rayyan (Qatar Computing Research Institute (Data Analytics), Doha, DH, Qatar) [61] to facilitate the title and abstract screening process. Titles and abstracts will be independently screened for eligibility using the inclusion criteria by two systematic reviewers (I.B. and S.L./K.C.). Studies identified as potentially eligible or those without an abstract will have their full text retrieved. The full texts of the studies will be assessed for eligibility by two independent reviewers. Full-text studies that do not meet the inclusion criteria will be excluded, citing reasons. Any disagreements that arise between the two reviewers will be resolved through discussion. If consensus is not reached, a third reviewer will be consulted. Translations will be sought where necessary.

### 2.4. Assessment of Methodological Quality

The included studies will be critically assessed by two independent systematic reviewers (I.B. and S.L./K.C.) for methodological quality using the standardised critical appraisal tool developed by JBI for RCTs [46]. This tool uses a series of criteria that can be scored as being met (yes), not met (no), unclear or not applicable (n/a). The two reviewers will independently assess each criterion and comment on it. Any disagreements that arise between the two reviewers will be resolved through discussion. If consensus is not reached, then a third reviewer will be involved. All studies, regardless of their methodological quality, will undergo data extraction and synthesis, where possible.

### 2.5. Data Extraction

Two systematic reviewers (I.B. and S.L./K.C.) will independently extract data from the included studies using a pre-developed and pre-tested data extraction form. Any disagreements that arise between the two reviewers will be resolved through discussion. If consensus is not reached, a third reviewer will be consulted. The following data will be extracted: author(s), year of publication, country, participant characteristics (e.g., age, sex, ethnicity, occupation and type of joints affected by osteoarthritis), sample size, intervention and comparator, outcomes (i.e., pain and function measurements), the timing of follow-up at the end of the intervention and adverse events. For both the outcomes, the authors will extract the end of intervention data [51,62]. Where this time point is not reported, data from the time point closest to the end of the intervention will be extracted. Intention-to-treat (ITT) data will be preferred compared to per-protocol data. ITT analysis is the most preferred analysis method for RCTs [63]. This is because it preserves sample size by including all the participants irrespective of adherence to the study and attrition, maximises external validity and helps in understanding real-world circumstances encountered in an actual setting [63,64]. Post-intervention data will be extracted in preference to change from baseline data (i.e., post-intervention score–baseline score). Percentage change from baseline will not be extracted, as it is highly sensitive to change in variance, and it also fails to protect from baseline imbalances, leading to non-normally distributed outcome data [65]. In addition, the content of yoga interventions will be extracted (e.g., yogic poses, breathing practices, meditation and relaxation practices), along with the structure (e.g., duration of the yoga sessions, and duration and frequency of the yoga interventions) and delivery characteristics (e.g., individual or group sessions, supervised or unsupervised sessions, sessions delivered in yoga centres or at home, strategies for yoga intervention uptake and adherence, and characteristics of yoga instructors).

To obtain missing data on outcomes, multiple strategies will be used. The first strategy will be to contact the corresponding author of the included study by email (at least two times per author) to obtain the relevant data. If we receive no response from the corresponding author, then certain assumptions will be applied. For example, where pain and function are reported as continuous outcomes, if the standard deviation (SD) is missing, SD will be imputed from a similar study (in terms of intervention, comparator, sample size and numerical outcome data) [66]. If only a median and interquartile range (IQR) is reported, these will be extracted, the mean will be assumed to be equal to the median, and the SD will be calculated using the standard formula (=IQR/1.35) [66]. 

### 2.6. Data Synthesis

A narrative synthesis will be conducted with the aid of tables and text, focusing on the content, structure and delivery characteristics of effective yoga interventions for the management of osteoarthritis (i.e., for each type of joint and outcome). For example, in the case of knee osteoarthritis, a narrative synthesis will be performed for knee pain and knee function.

Considering the errors in how authors analyse and report yoga interventions to be effective in studies (e.g., conducting pre-post analysis of outcomes within study arms but no comparative analysis between study arms), meta-analyses will be conducted for each type of joint and outcome using Review Manager 5.4.1 (Copenhagen, The Nordic Cochrane Centre, The Cochrane Collaboration) [67] to determine the true effectiveness of each included yoga intervention. Random-effects meta-analyses will be conducted to provide a weighted measure of treatment effect. For studies with more than one comparator group, the comparisons will be included in separate meta-analysis models to avoid the issue of double-counting of the comparator group. Where pain and function are reported as continuous outcomes, MDs with 95% CIs will be reported where the same scale is used across the studies. Where different scales are used across studies, standardised mean differences (SMDs) with 95% CIs will be reported. Where necessary, post-intervention data will be pooled with changes from baseline data, and this will be carried out for MDs but not SMDs. If reported as binary outcomes, risk ratios with 95% CIs will be reported.

In the final step, a meta-regression will be conducted to explore the statistical evidence for which the components of the intervention are effective [68]. This requires a reasonable number of studies in order to have sufficient power to show differences in effectiveness between components, and so the final decision on which components will be explored and how they will be grouped will be made once we have extracted data from the included studies on the components of each intervention. However, we anticipate exploring broad categories of intervention components, including content (e.g., yogic poses, breathing exercises, meditation and relaxation practices), structure (e.g., number of yoga sessions per week and length of the yoga sessions) and delivery characteristics (e.g., one-to-one or group sessions) of yoga interventions [68]. A random-effects model will be used to analyse these subgroup effects [69]. The effects of content, structure and delivery components on outcomes, i.e., pain and function, will be investigated by looking at the amount of heterogeneity explained by these components, using the reduction in the I^2^ statistic and the DerSimonian–Laird estimation method [70]. The results will establish the statistical significance of any observed patterns in which the components of yoga are associated with a greater effect on osteoarthritis outcomes.

## Appendix A. Search Strategies

**MEDLINE (Ovid) <1946 to 21 January 2022>: 190 records**

1. exp Mind-Body Therapies/
2. mind body therap*.mp.
3. meditat*.mp.
4. (yoga* or yogi*).mp.
5. (Asana* or Pranayam* or Dhyan* or Ashtanga or Bikram or Hatha or Iyengar or Kripalu or Kundalini or Vinyasa or Raja or Radja or Bhakti or Jnana or Kriya* or Karma or Yama or Niyama or Pratyahara or Dharana or Samadhi or Bandha or Mudra* or Chanda or Sivananda).mp.
6. 1 or 2 or 3 or 4 or 5
7. exp Osteoarthritis/
8. (osteoarthr* or osteo-arthr*).mp.
9. Coxarthrosis.mp.
10. Arthrosis deformans.mp.
11. (Degenerative adj2 arthritis).mp.
12. Degenerative joint disease*.mp.
13. Non-inflammatory arthritis.mp.
14. 7 or 8 or 9 or 10 or 11 or 12 or 13
15. randomized controlled trial.pt.
16. controlled clinical trial.pt.
17. randomized.ab.
18. placebo.ab.
19. drug therapy.fs.
20. randomly.ab.
21. trial.ab.
22. groups.ab.
23. 15 or 16 or 17 or 18 or 19 or 20 or 21 or 22
24. exp animals/ not humans.sh.
25. 23 not 24
26. 6 and 14 and 25

**EMBASE (Ovid) <1974 to 21 January 2022>: 454 records**

1. exp alternative medicine/
2. exp yoga/
3. exp meditation/
4. Mind body therap*.mp.
5. (Yoga* or yogi*).mp.
6. Meditat*.mp.
7. (Asana* or Pranayam* or Dhyan* or Ashtanga or Bikram or Hatha or Iyengar or Kripalu or Kundalini or Vinyasa or Raja or Radja or Bhakti or Jnana or Kriya* or Karma or Yama or Niyama or Pratyahara or Dharana or Samadhi or Bandha or Mudra* or Chanda or Sivananda).mp.
8. 1 or 2 or 3 or 4 or 5 or 6 or 7
9. exp osteoarthritis/
10. (osteoarthr* or osteo-arthr*).mp.
11. Coxarthrosis.mp.
12. Arthrosis deformans.mp.
13. (Degenerative adj2 arthritis).mp.
14. Degenerative joint disease*.mp.
15. Non-inflammatory arthritis.mp.
16. 9 or 10 or 11 or 12 or 13 or 14 or 15
17. Randomized controlled trial/
18. Controlled clinical trial/
19. random*.ti,ab.
20. randomization/
21. intermethod comparison/
22. placebo.ti,ab.
23. (compare or compared or comparison).ti.
24. ((evaluated or evaluate or evaluating or assessed or assess) and (compare or compared or comparing or comparison)).ab.
25. (open adj label).ti,ab.
26. ((double or single or doubly or singly) adj (blind or blinded or blindly)).ti,ab.
27. double blind procedure/
28. parallel group*1.ti,ab.
29. (crossover or cross over).ti,ab.
30. ((assign* or match or matched or allocation) adj5 (alternate or group*1 or intervention*1 or patient*1 or subject*1 or participant*1)).ti,ab.
31. (assigned or allocated).ti,ab.
32. (controlled adj7 (study or design or trial)).ti,ab.
33. (volunteer or volunteers).ti,ab.
34. human experiment/
35. trial.ti.
36. 17 or 18 or 19 or 20 or 21 or 22 or 23 or 24 or 25 or 26 or 27 or 28 or 29 or 30 or 31 or 32 or 33 or 34 or 35.
37. (random* adj sampl* adj7 (cross section* or questionnaire*1 or survey* or database*1)).ti,ab. not (comparative study/ or controlled study/ or randomi?ed controlled.ti,ab. or randomly assigned.ti,ab.)
38. Cross-sectional study/ not (randomized controlled trial/ or controlled clinical study/ or controlled study/ or randomi?ed controlled.ti,ab. or control group*1.ti,ab.)
39. (((case adj control*) and random*) not randomi?ed controlled).ti,ab.
40. (Systematic review not (trial or study)).ti.
41. (nonrandom* not random*).ti,ab.
42. Random field*.ti,ab.
43. (random cluster adj3 sampl*).ti,ab.
44. (review.ab. and review.pt.) not trial.ti.
45. we searched.ab. and (review.ti. or review.pt.)
46. update review.ab.
47. (databases adj4 searched).ab.
48. (rat or rats or mouse or mice or swine or porcine or murine or sheep or lambs or pigs or piglets or rabbit or rabbits or cat or cats or dog or dogs or cattle or bovine or monkey or monkeys or trout or marmoset$1).ti. and animal experiment/
49. Animal experiment/ not (human experiment/ or human/)
50. 37 or 38 or 39 or 40 or 41 or 42 or 43 or 44 or 45 or 46 or 47 or 48 or 49
51. 36 not 50
52. 8 and 16 and 51

**PsycInfo (OVID) <1806 to 21 January 2022>: 8 records**

1. exp Mind Body Therapy/
2. Mind-body therap*.mp.
3. exp Yoga/
4. (Yoga* or yogi*).mp.
5. exp Meditation/
6. Meditat*.mp.
7. (Asana* or Pranayam* or Dhyan* or Ashtanga or Bikram or Hatha or Iyengar or Kripalu or Kundalini or Vinyasa or Raja or Radja or Bhakti or Jnana or Kriya* or Karma or Yama or Niyama or Pratyahara or Dharana or Samadhi or Bandha or Mudra* or Chanda or Sivananda).mp.
8. 1 or 2 or 3 or 4 or 5 or 6 or 7
9. (Osteoarthr* or osteo-arthr*).mp.
10. Coxarthrosis.mp.
11. Arthrosis deformans.mp.
12. (Degenerative adj2 arthritis).mp.
13. Degenerative joint disease.mp.
14. Non-inflammatory arthritis.mp.
15. 9 or 10 or 11 or 12 or 13 or 14
16. (Randomized Controlled Trial or Controlled Clinical Trial or Pragmatic Clinical Trial or Equivalence Trial or Clinical Trial, Phase III).pt.
17. Randomized Controlled Trial/
18. exp Randomized Controlled Trials/
19. “Randomized Controlled Trial (topic)”/
20. Controlled Clinical Trial/
21. Controlled Clinical Trials/
22. exp Clinical Trials/
23. “Controlled Clinical Trial (topic)”/
24. Randomization/
25. Random Allocation/
26. Double-Blind Method/
27. Double Blind Procedure/
28. Double-Blind Studies/
29. Single-Blind Method/
30. Single Blind Procedure/
31. Single-Blind Studies/
32. Placebos/
33. Placebo/
34. Control Groups/
35. Control Group/
36. (random* or sham or placebo*).ti,ab,hw.
37. ((singl* or doubl*) adj (blind* or dumm* or mask*)).ti,ab,hw.
38. ((tripl* or trebl*) adj (blind* or dumm* or mask*)).ti,ab,hw.
39. (control* adj3 (study or studies or trial* or group*)).ti,ab.
40. (Nonrandom* or non random* or non-random* or quasi-random* or quasirandom*).ti,ab,hw.
41. allocated.ti,ab,hw.
42. ((open label or open-label) adj5 (study or studies or trial*)).ti,ab,hw.
43. ((equivalence or superiority or non-inferiority or noninferiority) adj3 (study or studies or trial*)).ti,ab,hw.
44. (pragmatic study or pragmatic studies).ti,ab,hw.
45. ((pragmatic or practical) adj3 trial*).ti,ab,hw.
46. ((quasiexperimental or quasi-experimental) adj3 (study or studies or trial*)).ti,ab,hw.
47. (phase adj3 (III or “3”) adj3 (study or studies or trial*)).ti,hw.
48. 16 or 17 or 18 or 19 or 20 or 21 or 22 or 23 or 24 or 25 or 26 or 27 or 28 or 29 or 30 or 31 or 32 or 33 or 34 or 35 or 36 or 37 or 38 or 39 or 40 or 41 or 42 or 43 or 44 or 45 or 46 or 47
49. 8 and 15 and 48

***CINAHL (EBSCOHost)* <1994 to 21 January 2022>: 288 records**

S1 (MH “Yoga”)
S2 (MH “Mind Body Techniques+”)
S3 (MH “Meditation”)
S4 TX Yoga* or yogi*
S5 TX “Mind body therap*”
S6 TX Meditat*
S7 TX Asana* or Pranayam* or Dhyan* or Ashtanga or Bikram or Hatha or Iyengar or Kripalu or Kundalini or Vinyasa or Raja or Radja or Bhakti or Jnana or Kriya* or Karma or Yama or Niyama or Pratyahara or Dharana or Samadhi or Bandha or Mudra* or Chanda or Sivananda
S8 TX S1 or or S2 or S3 or S5 or S6 or S7
S9 MH Osteoarthritis
S10 TX Osteoarthr* or Osteo-arthr*
S11 TX Arthrosis deformans
S12 TX Coxarthrosis
S13 TX Degenerative adj2 arthritis
S14 TX Degenerative arthritis
S15 TX Degenerative joint disease*
S16 TX Non-inflammatory arthritis
S17 TX S9 or S10 or S11 or S12 or S13 or S14 or S15 or S16
S18 (MH “Randomized Controlled Trials”)
S19 (MH “Double-Blind Studies”)
S20 (MH “Single-Blind Studies”)
S21 (MH “Random Assignment”)
S22 (MH “Pretest-Posttest Design”)
S23 (MH “Cluster Sample”)
S24 TI (randomised or randomized)
S25 AB (random*)
S26 TI (trial)
S27 TI MH (sample size) and AB (assigned OR allocated OR control)
S28 MH placebos
S29 PT randomized controlled trial
S30 AB control W5 group
S31 AB MH (crossover design) or MH (comparative studies)
S32 AB cluster W3 RCT
S33 MH animals+
S34 MH animal studies
S35 TI animal model*
S36 S33 or S34 or S35
S37 MH human
S38 S36 not S37
S39 S18 or S19 or S20 or S21 or S22 or S23 or S24 or S25 or S26 or S27 or S28 or S29 or S30 or S31 or S32
S40 S39 not S38
S41 S8 and S17 and S40

**Cochrane Central Register of Controlled Trials (CENTRAL) <1996 to 21 January 2022>: 269 records**

#1 MeSH descriptor: [Mind-Body Therapies] explode all trees
#2 MeSH descriptor: [Meditation] explode all trees
#3 (yoga*) (Word variations have been searched)
#4 (Mind body therapies) (Word variations have been searched)
#5 (yogi*) (Word variations have been searched)
#6 (asana* or pranayam* or dhyan* or meditat* or ashtanga or bikram or hatha or iyengar or kripalu or kundalini or vinyasa or raja or radja or bhakti or jnana or kriya* or karma or yama or niyama or pratyahara or dharana or samadhi or bandha or mudra* or chanda or sivananda) (Word variations have been searched)
#7 #1 or #2 or #3 or #4 or #5 or #6
#8 MeSH descriptor: [Osteoarthritis] explode all trees
#9 (osteoarthr*) (Word variations have been searched)
#10 MeSH descriptor: [Osteoarthritis, Hip] explode all trees
#11 (“arthrosis deformans”) (Word variations have been searched)
#12 Degenerative adj2 arthritis
#13 (“degenerative joint disease”) (Word variations have been searched)
#14 (“degenerative arthritis”) (Word variations have been searched)
#15 (non-inflammatory arthritis) (Word variations have been searched)
#16 #8 or #9 or #10 or #11 or #12 or #13 or #14 or #15
#17 MeSH descriptor: [Randomized Controlled Trial] explode all trees
#18 MeSH descriptor: [Controlled Clinical Trial] explode all trees
#19 placebo*
#20 (“randomised clinical trial”) (Word variations have been searched)
#21 (trial*) (Word variations have been searched)
#22 MeSH descriptor: [Randomized Controlled Trials as Topic] explode all trees
#23 #17 or #18 or #19 or #20 or #21 or #22
#24 #7 and #16 and #23

**Allied and Complementary Medicine (AMED) (Ovid) <1985 to 21 January 2022>: 10 records**

1. exp Mind body medicine/
2. exp Yoga/
3. exp Meditation/
4. Mind body medicine*.mp.
5. (Yoga* or yogi*).mp.
6. Meditat*.mp.
7. (Asana* or Pranayam* or Dhyan* or Ashtanga or Bikram or Hatha or Iyengar or Kripalu or Kundalini or Vinyasa or Raja or Radja or Bhakti or Jnana or Kriya* or Karma or Yama or Niyama or Pratyahara or Dharana or Samadhi or Bandha or Mudra* or Chanda or Sivananda).mp.
8. 1 or 2 or 3 or 4 or 5 or 6 or 7
9. exp Osteoarthritis/
10. (osteoarthr* or osteo-arthr*).mp.
11. Coxarthrosis.mp.
12. Arthrosis deformans.mp.
13. (Degenerative adj2 arthritis).mp.
14. Degenerative joint disease*.mp.
15. Non-inflammatory arthritis.mp.
16. 9 or 10 or 11 or 12 or 13 or 14 or 15
17. (random* or factorial* or placebo* or assign* or allocat* or crossover*).tw.
18. (cross adj over*).tw.
19. (trial* and (control* or comparative)).tw.
20. ((blind* or mask*) and (single or double or triple or treble)).tw.
21. (treatment adj arm*).tw.
22. (control* adj group*).tw.
23. (phase adj (III or three)).tw.
24. (versus or vs).tw.
25. rct.tw.
26. RANDOM ALLOCATION/
27. DOUBLE BLIND METHOD/
28. placebos/
29. randomized controlled trials/
30. 17 or 18 or 19 or 20 or 21 or 22 or 23 or 24 or 25 or 26 or 27 or 28 or 29
31. 8 and 16 and 30

**SPORTDiscus (EBSCOHost) <2004 to 21 January 2022>: 12 records**

1. SU Mind body therapy
2. SU Yoga
3. SU Meditation
4. TX Mind body therap*
5. TX Yoga* or yogi*
6. TX Meditat*
7. TX Asana* or Pranayam* or Dhyan* or Ashtanga or Bikram or Hatha or Iyengar or Kripalu or Kundalini or Vinyasa or Raja or Radja or Bhakti or Jnana or Kriya* or Karma or Yama or Niyama or Pratyahara or Dharana or Samadhi or Bandha or Mudra* or Chanda or Sivananda
8. TX S1 or S2 or S3 or S4 or S5 or S6 or S7
9. SU Osteoarthritis
10. TX Osteoarthr* or osteo-arthr*
11. TX Coxarthrosis
12. TX Arthrosis deformans
13. TX degenerative arthritis
14. TX degenerative joint disease*
15. TX Non-inflammatory arthritis
16. TX S9 or S10 or S11 or S12 or S13 or S14 or S15
17. SU Randomized controlled trials
18. TX Randomized controlled trials
19. TX Double-blind studies
20. TX Single-blind studies
21. TX Random assignment
22. TX Pretest-posttest design
23. TX Cluster sample
24. TX Placebos
25. TX randomised or randomized
26. TX random*
27. TX trial*
28. TX S17 or S18 or S19 or S20 or S21 or S22 or S23 or S24 or S25 or S26 or S27
29. (TX S17 or S18 or S19 or S20 or S21 or S22 or S23 or S24 or S25 or S26 or S27) and (S8 and S16 and S28)

**Web of Science <1998 to 21 January 2022>: 125 records**

#1 ALL= (yoga* or “mind body therap*” or meditation or yogi* or asana* or pranayam* or dhyan* or meditat* or ashtanga or bikram or hatha or iyengar or kripalu or kundalini or vinyasa or raja or radja or bhakti or jnana or kriya* or karma or yama or niyama or pratyahara or dharana or samadhi or bandha or mudra* or chanda or sivananda)
#2 ALL= (“Osteoarthritis” or osteoarthr* or osteo-arthr* or “coxarthrosis” or degenerative arthritis or degenerative joint disease* or non-inflammatory arthritis)
#3 ALL= (“randomized controlled trial” or “controlled clinical trial” or “clinical trial” or “clinical
trials” or placebo$ or “random allocation” or “double-blind method” or “single-blind
method” or “cross-over studies”)
#4 TS= (randomised or randomized or randomisation or randomisation or
placebo* or (random* and (allocat* or assign*)) or (blind* and
(single or double or treble or triple)))
#5 #3 or #4
#6 #1 and #2 and #5

**Turning Research Into Practice (TRIP); < 2014 to 21 January 2022>: 350 records**

(yoga* or “mind body therapies” or yogi* or asana* or pranayam* or dhyan* or meditation or meditate or ashtanga or bikram or hatha or iyengar or kripalu or kundalini or vinyasa or raja or radja or bhakti or jnana or kriya* or karma or yama or niyama or pratyahara or dharana or samadhi or bandha or mudra* or chanda or sivananda) and (“osteoarthritis” or osteoarthr* or osteo-arthr* or coxarthrosis or degenerative arthritis or degenerative joint disease* or non-inflammatory arthritis) and (randomised controlled trial or randomized controlled trial or controlled clinical trial* or clinical trial* or placebo* or random*)

**AYUSH Research Portal (Ministry of AYUSH, Government of India) <Date of search: 21 January 2022>: 33 records**

Medical system: yoga and naturopathy
Category: preclinical research, clinical research (Evidence grade-A, B, C) and fundamental research
Body system: musculoskeletal
Disease (English): Osteoarthrosis of hip (ICPC-L89) (ICD-M16)

Evidence grade- A:1 Evidence grade-B:0 Evidence grade-C:0

Disease (English): osteoarthrosis of knee (ICPC-L90) (ICD-M17)
Evidence grade-A:6 Evidence grade-B:23 Evidence grade-C:0

Disease (English): osteoarthrosis other (ICPC-L91) (ICD-M13, M15, M18, M19)
Evidence grade-A:1 Evidence grade-B:2 Evidence grade-C:0

**A Bibliography of Indian Medicine (ABIM) < Date of search: 21 January 2022>: 1 record**

Search terms used: yoga and osteoarthritis

**Complementary and alternative medicine (CAM-QUEST) < Date of search: 21 January 2022>: 61 records**

Searched for the following:
Disease pattern: pain (1155 results) to disease: chronic pain (209 results) to therapy: mind body medicine (75 results) to study design: randomized trial (33 results)

Searched for the following:
Therapy: Mind-body medicine (3708 results) to disease pattern: Musculoskeletal-/Connective tissue system (388 results) to disease: Arthritis (24 results) to study design: Randomized trial (13 results)

Therapy: Mind-body medicine (3708 results) to disease pattern: Musculoskeletal-/Connective tissue system (388 results) to disease: Arthrosis (37 results) to study design: Randomized trial (15 results)

**Physiotherapy Evidence Database (PeDro) <1999 to 22 January 2022>: 19 records**

Searched terms used: yoga and osteoarthritis
39 records out of which 19 RCTs

**OpenGrey (Data Archiving and Network Services) <1997 to 21 January 2022>: 0 records**

0 results for yoga and osteoarthritis

**EthOS <2009 to 21 January 2022>: 0 records**

0 results for yoga and osteoarthritis and trials

**ProQuest Dissertations and Theses <1902 to 21 January 2022>: 286 records**

(yoga* or mind body therap* or yogi* or asana* or pranayam* or dhyan* or meditat* or ashtanga or bikram or hatha or iyengar or kripalu or kundalini or vinyasa or raja or radja or bhakti or jnana or kriya* or karma or yama or niyama or pratyahara or dharana or samadhi or bandha or mudra* or chanda or sivananda) and (osteoarthritis or osteoarthr* or osteo-arthr* or coxarthrosis or degenerative adj2 arthritis or degenerative joint disease* or non-inflammatory arthritis) and (randomised controlled trial or randomized controlled trial or controlled clinical trial* or clinical trial* or placebo* or random* or trial*)

Filters applied: Osteoarthritis or arthritis

**DART-Europe-e-theses portal <2005 to 21 January 2022>: 0 records**

0 results for yoga and osteoarthritis
